# Progress in Polymeric Nano-Medicines for Theranostic Cancer Treatment

**DOI:** 10.3390/polym12030598

**Published:** 2020-03-06

**Authors:** Imran Ali, Mosa Alsehli, Luciana Scotti, Marcus Tullius Scotti, Shang-Ting Tsai, Ruei-Siang Yu, Ming Fa Hsieh, Jung-Chih Chen

**Affiliations:** 1 Department of Chemistry, College of Sciences, Taibah University, Al-Medina Al-Munawara 41477, Saudi Arabia; mhsehli@taibahu.edu.sa; 2 Department of Chemistry, Jamia Millia Islamia (Central University), New Delhi 110025, India; 3 Cheminformatics Laboratory—Postgraduate Program in Natural Products and Synthetic Bioactive, Federal University of Paraíba-Campus I, João Pessoa 58051-970, PB, Brazil; luciana.scotti@gmail.com (L.S.); mtscotti@gmail.com (M.T.S.); 4 Department of Biomedical Engineering, Chung Yuan Christian University, 200 Chung Pei Road, Chung Li District, Taoyuan 32023, Taiwan; shant7707@gmail.com (S.-T.T.); shung804@yahoo.com.tw (R.-S.Y.); mfhsieh@cycu.edu.tw (M.F.H.); 5 Center for Minimally-Invasive Medical Devices and Technologies, Chung Yuan Christian University, 200 Chung Pei Road, Chung Li District, Taoyuan 32023, Taiwan; 6 Department of Pharmacy, Kaohsiung Armed Forces General Hospital, No.2, Zhongzheng 1st Rd., Lingya Dist., Kaohsiung 80284, Taiwan; 7 Institute of Biomedical Engineering, National Chiao Tung University, 1001 University Rd., Hsinchu 300, Taiwan; george@nctu.edu.tw

**Keywords:** theranostic cancer treatment, polymeric nanoparticles, merits and demerits, approved nano-medicines, future perspectives

## Abstract

Cancer is a life-threatening disease killing millions of people globally. Among various medical treatments, nano-medicines are gaining importance continuously. Many nanocarriers have been developed for treatment, but polymerically-based ones are acquiring importance due to their targeting capabilities, biodegradability, biocompatibility, capacity for drug loading and long blood circulation time. The present article describes progress in polymeric nano-medicines for theranostic cancer treatment, which includes cancer diagnosis and treatment in a single dosage form. The article covers the applications of natural and synthetic polymers in cancer diagnosis and treatment. Efforts were also made to discuss the merits and demerits of such polymers; the status of approved nano-medicines; and future perspectives.

## 1. Introduction

**Figure 1:**
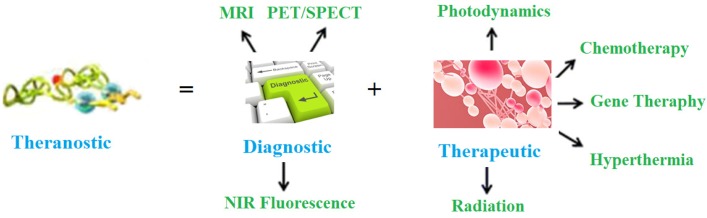
The schematic representation of polymeric nano-partcles (NPs) theranostic approach.

The cancer is a complex, heterogeneous, aggressive and horrible disease, killing millions of people globally. As per *GLOBOCAN* estimation, 18.1 million new cases and 9.6 million deaths occurred worldwide in 2018 due to cancer alone [1]. Therefore, there is a great need to combat this deadly disease. Nowadays, theranostic management of various diseases is gaining importance, including horrible cancer ailments. The term theranostic was given by John Funkhouser (US consultant) in August, 1998 in a press release from the company Cardiovascular Diagnostics. It was used to combine investigative and treatment approaches into a single unit. It involves a set of diagnoses, a drug carrier and the observation of treatment reaction. This approach provides treatment protocols that are more precise to individuals, and hence, are more likely to deliver better predictions by diagnosis and treatment. It delivers exact, safe and targeted treatment via the right drug, in the right dose and at the right place in the human body. Besides, theranostic management also contributes to the economy of a country by reduced cost strategies with specific and efficient medicine procedures. It is a targeted treatment protocol, and hence, has very little side effects. This approach is represented in Figure 1.

The number of publications in polymer therapeutics is increasing continuously. During our research of more than 30 years and available literature on cancer treatment [2,3,4,5,6,7,8,9,10,11,12,13,14,15], it was observed that chemotherapy is considered the best approach to curb different types of cancers [16,17,18,19,20,21,22,23,24,25,26,27,28]. But this has severe toxicity to normal cells. It is a challenge to researchers, academicians and oncologists. Therefore, a theranostic approach may be useful for treating cancer, and of course, gaining efficacy for patients.

In cancer treatment and management, the presently used techniques are not capable of diagnosing cancers in early stages and are also not very specific. However, nanotechnology is providing a bias for the diagnosis, treatment and management of different cancers [29,30]. The nanotechnology is offering a chance for diagnosis and therapy for cancer. Nanoparticle-centered imaging and therapy are being investigated continuously. The nanotechnology is proficient in diagnosis, drug delivery and observation of therapeutic response. It is likely to perform an important role in personalized medicine and treatment. The polymeric nanoparticles, metallic nanoparticles, liposomes, dendrimers, carbon nanotubes quantum dots, etc., are being used as nano-formulations for cancer theranostics. Among these nanoparticles, polymeric ones are gaining importance due to their ease of preparation, high drug-loading capacities, their biocompatibility and biodegradable nature. Much work has been carried out in this direction and some reviews are available on the subject [31,32,33,34,35,36,37,38], but there is no review specially dedicated to polymeric nana-particles (NPs) in the diagnoses and treatments of various cancers. Therefore, this is the time to review the progress in polymeric nano-medicines for theranostic cancer management. The present article describes a state-of-the-art of polymeric nano-medicines for theranostic cancer management. Besides, effort was made to highlight future challenges and perspectives in this area of research.

## 2. Nanoparticles

**Figure 2:**
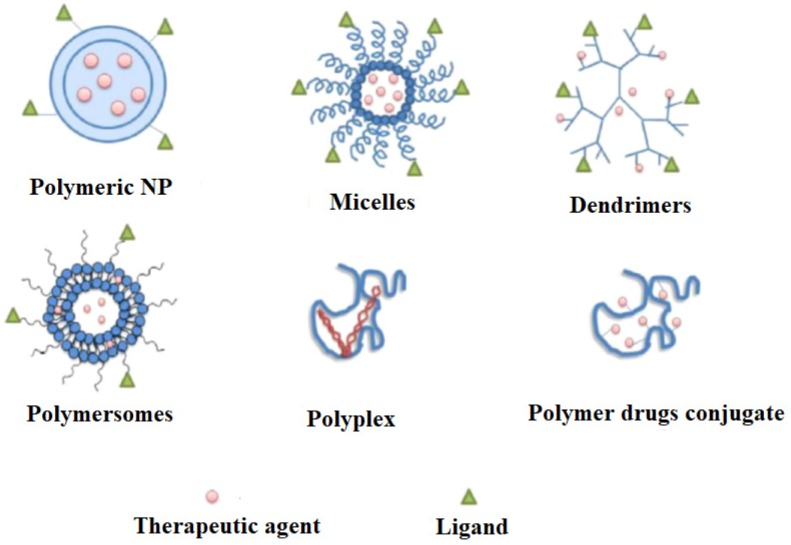
The different types of polymeric NPs as carriers for drugs delivery.

**Figure 3:**
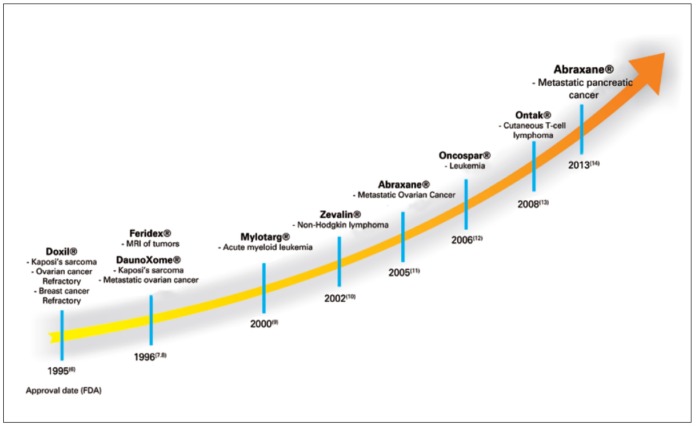
The timeline of the development of polymeric NPs based drugs for cancer treatment [79].

Nano theranostic is a growing field for instantaneous diagnosis, drug delivery and curing cancers [39,40,41]. It is well known that nanoparticles have diameters ranging from 1.0 to 100.0 nm, which are about a thousand times smaller than the normal human cells. Besides, these nanoparticles have great chances of cellular interactions with cellular receptors, enzymes and antibodies [42]. The facility of nanoparticle modification makes them ideal candidates for the exact diagnosis and treatment [43,44]. The literature indicates the formulations of several theranostic nanoparticle agents. These are made of several materials, including ions of gold, silica carbon, etc. [45,46]. These were evaluated in many animal models, and have found promising identities in early detection of cancer via several imaging probes. But the drawbacks associated with them are immunogenicity, toxicity and slow excretion rate from the body [47,48]. Accordingly, some macromolecules have been tested for these purposes. The most important molecules exploited for such platforms are polylactic acid (PLA), poly(ε-caprolactone), poly(lactide-co-glycolide) (PLGA), poly(alkylcyanoacrylate) and polyglycolic acid. Contrarily, the natural polymers used are peptides, proteins, nucleic acids, dextran ester and chitosan. Of course, these molecules are excellent but suffer from short half-lives, non-specific distribution rates and limited applications because of their interactions with drug molecules. Consequently, synthetic, polymeric, biodegradable nanoparticles were explored [49,50]. These polymeric nanoparticles were developed with much effort through simulation studies and synthetic chemistry. The various synthetic biodegradable polymeric nanocarriers are poly(2-hydroxyethyl-L-aspartamide), poly(L-aspartate), poly(D,L-lactic acid-co-glycolic acid), poly(ε-caprolactone), poly(ethylene glycol) (PEG), poly(N-vinyl pyrrolidone) (PVP), poly(N-isopropyl acrylamide) (PNIPAM), poly(hydroxypropyl methacrylamide) (PHPMA), poly(methyl methacrylate), poly(ethylene glycol), poly-(chloromethyl-styrene) (PCMS), etc. The theranostic cancer management is based on diagnosis and treatment, and hence, this review is divided into two parts; i.e., diagnosis and treatment of cancer. The different types of polymeric NPs for drugs delivery are shown in Figure 2. The timeline of the development of polymeric NPs for cancer treatment is shown in Figure 3. The biocompatible polymer-based cancer theranostics are given in Table 1, while polymeric NPs in clinical trials are given in Table 2.

## 3. Diagnosis of Cancer

The early detection of cancer is a very important issue in order to treat this disease properly. Cancers can be treated and managed if diagnosed in an early stage. In this way, the early diagnosis of cancer is one of the most important approaches to manage cancers. Unluckily, only 16% of cancer cases are diagnosed prior to malignancy and most of them are noticed in advanced or metastatic or late stages [80]. Generally, the diagnoses of cancers depend on the type of cancer, and sizes and positions of tumors. The commonly used ways of cancer diagnosis are biopsy, positron emission tomography scanning, magnetic resonance imaging, computed tomography, biosensors, X-rays, radionuclide, fluorescent imaging, etc. The nanotechnology approach has been coupled to the above modalities to detect cancers in the early stage. This section deals with the diagnosis of cancer using polymeric NPs only. Many NPs have been used to detect cancers but polymeric-based ones are considered the best one due to biodegradable nature and capacity to carry many lesion-detecting molecules. Besides, polymeric NPs have targeting moieties that show a therapeutic drug with an imaging agent to be expected to have an excellent theranostic platform. Additionally, polymeric NPs may directly transport the imaging agent to the lesion, and can be simply administrated by inhalation, oral or intravenous ways, respectively. The most common identities used for cancer diagnosis are cancer antigen 125 (CA125), α-fetoprotein (AFP), cancer antigen 19-9 (CA19-9), cancer antigen 153 (CA15-3), carcinoembryonic antigen (CEA), epidermal growth factor receptor (EGFR), breast cancer (BRCA), prostate-specific antigen (PSA), interleukins (ILs), mucin 1 (MUC 1), tumor necrosis factor-alpha (TNF-α), human epidermal growth factor receptor 2 (HER2), vascular endothelial growth factor (VEGF) and squamous cell carcinoma antigen (SCC-Ag). These are identified by means of nanotechnology. Generally, these are used to precisely transport imaging and therapeutic molecules (theranostic agents) into the sites of cancerous tissues.

The following are the essential requirements for NPs for cancer diagnosis:

1. Being biocompatible and biodegradable in nature;
2. Low toxicity to normal cells;
3. Small size;
4. High stability in physiological conditions;
5. Capacity to carry the imaging agents;
6. Controlled release of therapeutic agents.

Gadolinium NPs have been used to detect tumors in magnetic resonance tomography (MRI) because of their good contrast. Boyes et al. [81] reported a review addressing the chemical aspects in the development of molecular probes based upon gadolinium NPs and their potential role in translational clinical imaging and therapy. Similarly, Cao et al. [82] reviewed the applications of prepared gadolinium NPs for cancer detection in MRI. The authors described these NPs to have the promising capability of improved longitudinal relaxivity of individual gadolinium ions. Further, the authors highlighted the developments and applications with future perspectives for their future development. Mi et al. [83] reported gadolinium (Gd) chelates-loaded nanocarriers for achieving magnetic resonance imaging (MRI)-guided Gd neutron capture therapy (GdNCT) of tumors. The authors developed calcium phosphate micelles hybridized with PEG-polyanion block copolymers, and united them with MRI contrast agent Gd-diethylenetriaminepentaacetic acid (Gd-DTPA/CaP). The Gd-DTPA/CaP displayed an increased distribution of Gd-DTPA in tumors, leading to a sufficient MRI contrast improvement of tumor location. Vuu et al. [84] used gadolinium−rhodamine NPs, prepared from lipid monomers for cancer detection in MRI. As per the authors, these NPs might be used to track cells in vivo.

Some other important works in the direction of cancer diagnosis are described in the following sub-sections. Mitra et al. [85] reviewed the applications of liposomes, micelles, dendrimers and water-soluble polymers for cancer diagnosis. The authors used these NPs to carry large payloads, improving cancer detection. The authors used nuclear imaging techniques such as dual-modality imaging with positron emission tomography/computed tomography (PET/CT) to detect cancer in various models. As per the authors, furthermore pre-clinical, clinical and long-term toxicity studies are needed before applying in real-life problems. Janib et al. [86] and Park et al. [87] reviewed the theranostic NPs, including drug conjugates, drug complexes, micelles, dendrimers, core-shell particles and carbon nanotubes. A lot of these formulations were applied for carriers of drugs or contrast agents. To observe the interactions of these formulations with cancer disease, contrast agents, including metals, metal oxides, optically active molecules, radionuclides and ultrasonic contrast mediums, were encapsulated. Wang et al. [88] used poly(ethylene glycol) and ATP aptamer to detect breast cancer by estimating ATP levels in the biological samples. The authors reported detection limit 0.1 pM with a linear range of 0.1–1000 pM. Lipid-polymer hybrid (LPH) NPs have been utilized to diagnose cancers. These acts as imaging agents, which are used in tomography or magnetic imaging resonance (MRI). Aryal et al. [89] described the use of LPHs in MRI, the former having a shell of gadolinium ions chelated with lipid PEG and DOTA. These were used to test in-vitro uptake by J-774 cells while the murine xenograft (B16-F10) model indicated an accumulation of the LPNs in the tumor within a time of 3 h.

Kandel et al. [90] used LPHs having a fluorescent cores; e.g., poly-(fluorene-alt-benzothidiazole)-PFBT enclosed by a lipid-PEG (DMPE-PEG) layer. The prepared LPHs, having particle sizes of 20–30 nm and quantum yield of ≈50%, indicated bright fluorescence because of the insertion of lipid tail in polymeric core and resulted in an augment of space and intrachain quenching. Sun et al. [91] used gold NPs and SPION-loaded micelles for in vitro detection of glioblastoma multiforme (brain cancer). These NPs were used as a contrast agent in computed tomography and MRI. These results were used to determine tumor size. As per the authors, the reported NPs were excellent agents in MRI, even after few days. It was realized that these NPs may be used as MRI agents for early detection of brain cancer. Mottaghitalab et al. [92] prepared silk fibroin NPs (SFNPs) beset with SP5-52 peptide for supplying Gem into lung cancer cells. The NPs were found to accumulate in cancer parts. This confirmed the efficiency of the synthesized NPs in cancer detection. Guo et al. [93] prepared polymeric nanosturucture (PHEMA-star-PLLA-PEG) with poly(hydroxyethyl methacrylate) core and PLLA-PEG as arms for cancer diagnosis. 1,4,7-triazacyclononane-1,4,7-triacetic acid and TRC105 and were altered (grafted) on the end groups for CD105 facilitated target, as well as ^64^Cu labelling and PET imaging. In vivo imaging and assessments showed that targeted ligand awarded this particular polymer with long dispersed half-time, improved tumor increase and a decent image comparison. As per the authors, quick imaging might be obtained via this imaging policy, with tumors noticeable at 0.5 h post-injection.

Magnetic NPs are the best ones in MRI, as these provide good contrast. In this series, Li et al. [94] prepared aqueous dispersive polyethyleneimine (PEI) coated Fe_3_O_4_ nanoparticles following modification by fluorescein isothiocyanate (FI) and PEGylated folic acid (FA) through PEI facilitated conjugation. The residual PEI surface amines were exposed to acetylation to make the colloidally stable FA functionalized Fe_3_O_4_ NPs for MRI. The authors claimed that the developed NPs may be used in vivo and in vitro for the diagnosis of different tumors. Wang et al. [95] prepared optical activated NPs for photoacoustic/fluorescent imaging in cancer diagnosis. The NPs consisted of perfluorohexane liquid and gold nanoparticles (GNPs) in the core stabilized by a poly(lactide-co-glycolic acid) (PLGA) polymer. The authors claimed that the reported NPs were evaluated in vitro experiments, showing the potential of photoacoustic imaging for future clinical investigations in cancer therapy. Huang et al. [96] designed polymeric substrate nanoparticles for cancer detection by the dissipative particle dynamics simulation. It was observed that the cancer cells and the normal cells were differentiated by different uptakes of nanoparticles. The antagonism between nanoparticle cell-specific and nanoparticle polymer non-specific interactions was the chief issue for dissimilar uptake performances. The authors enhanced cancer detection by investigating the effects of the polymer and ligand types, and density. As per the authors, this study may deliver valuable visions into the proposal of the functionalized substrate-based nanomaterials in biomedicine.

Ali et al. [97] used gold and polymeric superparamagnetic iron oxide nanoparticles (SPIONs) loaded micelles (GSMs) coated by polyethylene glycol-polycaprolactone (PEG-PCL) polymers for detecting brain tumors. GSMs were used as a contrast agent for both computed tomography (CT) and magnetic resonance imaging (MRI) studies of stereotactically implanted GBM tumors in a mouse model. These results suggested that with further expansion and testing, GSMs may possibly be combined into both imaging and treatment of brain tumors, helping a theranostic determination as both an MRI-based contrast agent and a radiosensitizer. The polymeric micelles have been used in nuclear imaging, X-rays, MRIs, and CT scans for cancer diagnosis [98]. Utilization of micelles conjugated with the gamma emitters (^99m^Tc and ^111^In) includes non-invasive biodistribution [99,100]. The addition to iron oxide particles in micelles or the use of chelators for the blending of paramagnetic metals to the hydrophilic wedge of micelle making block copolymers are two methods used for preparing micelle-based MRI contrast means [101,102,103,104,105]. The multifunctional polymeric micelles with imaging contrast means and bunches of magnetic NPs for effective MRI and chemotherapeutic agents targeting ligands have been described [105]. Qiao and Shi [106] and Yang et al. [107] described the syntheses of iron oxide NPs conjugated with Arg-Gly-Asp modified dendrimers for targeted MRI of C6 glioma cells.

## 4. Treatment of Cancer

Nanotechnology is gaining importance in treating and managing cancer. Different types of nanoparticles are used to control cancer diseases. Among many NPs, polymer-based NPs are gaining importance in cancer treatment. It is because these NPs are biocompatible, biodegradable, non-toxic and non-immunogenic. Different types of polymeric NPs, including composite NPs, have been used in cancer treatment, and these are discussed in the following sub-sections.

The salient requirements of a NP to treat and control a cancer are that it:

1. Should be biocompatible, biodegradable, non-toxic and non-immunogenic.
2. Should be able to carry the required drugs.
3. Should be able to discharge the medications at the location of the tumor.
4. NPs–drug complex should be stable under physiological conditions.
5. NPs–drug complex should be able to control either by receptor-facilitated interfaces or by electroparamegnetic resonance spectroscopy (EPR) effect.

As discussed above, polymeric nanoparticles (PNPs) are excellent carriers of the required drugs in cancer treatment. Many synthetic and natural precursors are being used for the preparation of polymeric NPS. The synthetic ones include poly(lactic-co-glycolic acid), poly(lactic acid) and polyethyleneimine, whereas the natural ones are albumin, collagen, chitosan and gelatin. First of all, in 1980, Couvreur et al. [108] used polymeric nanoparticles for cancer treatment. The authors developed PNPs by using poly(alkyl cyanoacrylate). After this research, many PNPs were prepared and used in curing cancer disease, especially the delivery of anti-cancer drugs. The applications of PNPs in cancer treatment are discussed in the following sub-sections.

### 4.1. Biodegradable Nanoparticles

The biodegradable polymeric nanoparticles are submicron-sized particles with drug molecules adsorbed, diffused, entangled, linked or encapsulated into the NPs or a gene of interest encapsulated inside a polymeric matrix. There are two methods for the preparation of nanoparticles: (i) by distributing polymers that are performed, and (ii) polymerizing monomers [109]. These NPs are easy to manufacture, relatively low-cost, biocompatible, non-immunogenic, non-toxic and water-soluble. These are also of great worth as an effective method for dispensing pharmaceuticals to particular tissues/organs, as a means of DNA gene therapy, and for their competency with distributing proteins, peptides and genes by oral administration [110]. The anticancer drugs, cisplatin, doxorubicin, paclitaxel, 5-fluorouracil, triptorelin, 9-nitrocamptothecin, xanthone, dexamethasone, etc., have been effectively related with poly(glycolic acid) (PLGA) [111]. The nanospheres having naturally degradable hydrophobic poly(ε-caprolactone) (PCL) and hydrophilic methoxy poly(ethylene glycol) (MePEG) have been used for taxol delivery [112].

### 4.2. Polymeric Superparamagnetic NPs

The magnetic NPs stay in superparamagnetic states, but have magnetic responses and super-paramagnetism. These NPs are used to enhance the therapeutic presentation of drugs and decrease the side effects related to the straight treatment of cancer. These may be guided towards the tumors with the help of outside magnetism. By binding these NPs with synthetic and natural polymers, they may be used as drug carriers. The polymeric superparamagnetic iron oxide nanoparticles (SPIONs) were coupled with polymersomes and micelles for drug delivery. This is due to the fact that these have good drug loading with biocompatibility, stability, fine particle size distribution, prolonged blood circulation and controlled drug release [43]. Huang et al. [113] described SPIONs coupled with folic acid for dual-targeted drug delivery. The doxorubicin (DOX) was laden onto folic acid (FA)-SPIONs nanoparticles covered with PEI (polyethylenimine) and PEG poly(ethylene glycol) polymers. As per the authors, the drug release was magnetically targeted and pH-sensitive. The prepared formulation was applied to the mice model with MCF-7 breast cancer. The authors reported an enhanced anticancer activity. Guthi et al. [114] used multifunctional micelle superparamagnetic iron oxide NPs for carrying doxorubicin and tested for H2009 lung cancer cells. The authors reported successful doxorubicin release. Maeng et al. [115] used superparamagnetic iron oxide to grow a drug delivery method with improved efficacy and minimized adverse effects by coating poly(ethylene oxide)-trimellitic anhydride chloride-folate and doxorubicin. The efficiency of the nanoparticles was assessed in rats and rabbits with liver cancer, in comparison with free-DOX (FD) and a commercial liposome drug, doxorubicin (DOXIL). As per the authors, these results indicated that the reported NPs may be good agents for curing liver cancer.

Ling et al. [116] used PLGA to encapsulate SPIONs and docetaxel for releasing the drugs to tumor cells. The multifunctional polymer vesicles were prepared by carboxy-terminated poly(lactide-co-glycolidelactide) utilizing a single emulsion evaporation method. The authors reported a 147 nm diameter with drug encapsulation efficacy of 6.02%–23%. The NPs showed a triphasic drug release pattern in vitro in 30 days. Improved cellular uptake ability and antiproliferative effect in prostate cancer PC3 cells were observed. Therefore, these stable and tumor targeting polymer NPs could be promising multifunctional vesicles for simultaneous targeting imaging, drug delivery and real-time monitoring of therapeutic effect. Yang et al. [117] used coated SPIONs for targeted drug delivery. The anticancer drug was conjugated onto the PEGylated SPIONs by pH-sensitive bonds. As per the authors, coated SPIONs indicated higher tumor accumulation than Arginylglycylaspartic acid (cRGD)-free ones. Thus, these coated SPIONs showed encouraging properties for combined targeted anticancer drug delivery. Yallapu et al. [118] synthesized superparamagnetic iron oxide nanoparticles by precipitation of iron salts in the presence of ammonia and β-cyclodextrin and pluronic polymer coatings. The curcumin-loaded formulation showed a good effect on ovarian, breast and prostate cancers. The better therapeutic effects were confirmed by molecular effects using western blotting and transmission electron microscopic (TEM) studies. Chen et al. [119] used copolymer of reducible polyamidoamine with polyethylene glycol/dodecyl amine for delivery of doxorubicin to targeted cells. Xu et al. [120] prepared multifunctional nanoparticles by using a superparamagnetic iron oxide core with PEG/PEI/polysorbate 80 (Ps 80), and that was utilized to encapsulate DOX. As per the authors, the NPs were capable of delivering drugs effectively to gliomas. Because of the magnetic targeting and Ps 80 arbitrated endocytosis; DOX@Ps 80-SPIONs in the occurrence of a magnetic field, led to the whole destruction of glioma growth in vivo at 28 days after treatment. The therapeutic mechanism of DOX@Ps 80-SPIONs acted by tempting apoptosis through the caspase-3 pathway.

### 4.3. Polymeric Gadolinium NPs

The polymeric gadolinium NPs have great potential in cancer diagnosis in MRI due to their good thermodynamic association constants and low dissociation kinetic rates. Besides, these NPs have been used as drug carriers for cancer treatment. Some of the important research works are summarized in this section. Lio et al. [121] used galodinium paramagnetic metal ions to prepare NPs with poly(DL-lactide-coglycolide; PLGA) and a hydrophilic paramagnetic folate coated PEGylated lipid shell (PFPL) having a core-shell structure. The NPs had good properties to load and release the hydrophobic drugs. As per the authors, the reported NPs had a good perspective on cancer treatment. Lee et al. [122] used Cd(III) NPs for the delivery of gemcitabine drugs. The Gd(III) conjugates indicated good drug uptake and control. Cheng et al. [123] reported polymeric (Gd2O3) nanoparticles with high longitudinal relaxation rate. The polymer used was polypyrrole (PPy) modified with hyaluronic acid (HA) and laden aluminum phthalocyanine (AlPc). The authors described the use of these NPS in MIR cancer detection and remote-controlled, PTT/PDT combined anti-tumor therapy. The nanoparticles showed a photothermal effect that may have triggered the release and de-quenching of AlPc.

Rowe et al. [124] prepared a multifunctional polymeric gadolinium (Gd) metal-organic framework (MOF) nanoparticles for cancer diagnosis and treatment. The polymer used was poly(N-isopropylacrylamide)-co-poly(N-acryloxysuccinimide)-co-poly(fluorescein O-methacrylate). Cao et al. [125] described diagnosis and therapy for cancer by developing magnetic nanoparticle (Gd@SiO2-DOX/ICG-PDC) integrating doxorubicin (DOX), indocyanine green (ICG) and gadolinium(III)-chelated silica nanospheres (Gd@SiO_2_) with a poly(diallyldimethylammonium chloride) (PDC) coating. The DOX release from Gd@SiO2-DOX/ICG-PDC depended on temperature and pH. As per the authors, the reported NPs are safe in both diagnosis (MRI scanning) and treatment. Roy et al. [126] reported the preparation of stimuli-responsive, polymer-adapted, gadolinium-doped iron oxide nanoparticles (poly@Gd-MNPs) and the use as cancer theranostic NPs. The NPs showed an excellent loading capacity for anti-cancer drug methotrexate along with stimuli dependent release by changing pH and temperature of the cancer cells. The poly@Gd-MNPs showed 86% drug loading capacity with 70% drug release in the first 2 h. The cytotoxic assay (MTT) established that the NPs did not disturb the feasibility of normal cells but capably killed the MCF7 cancer cells in the attendance of an outside magnetic field.

### 4.4. Polymer Gold NPs

Gold metal has been used for long to treat epilepsy and tuberculosis, and the properties of gold NPs make them ideal candidates. The chemical, surface, electronic and optical properties of gold NPs are unique for cancer treatment. These NPs may be delivered to cancer tissues by passive or active targeting plans. The various shapes of gold NPS are nanorods, nanoshells, nanospheres, nanostars, nanoboxes, nanocrystal, nanocages, nanoclusters, nanocubes and triangular bipyramaids. The modifications of these NPs with polymeric materials make them ideal candidates for drug delivery with increased circulation time, improved drugs-loading ability and stability and reduced cytotoxicity.

Gold NPs coagulated with polymers are ideal candidates for drug release due to the ease of surface modification. The surface modifications augment circulation time, drug targeting and the progressive stability of NPs, and decreases cell cytotoxicity. The chemotherapeutic anticancer drugs are loaded on these NPs via covalent and ionic bonds and physical sorption. The drugs loaded with these NPs are doxorubicin, methotrexate, paclitaxel, docetaxel, tamoxifen, oxaliplatin and 3-mercaptopropionic [127]. Ding et al. [128] conjugated paclitaxel with mercapto functionalized AuNPs using oligoethylene glycol spacer via carbodiimide based esterification for improved cytotoxicity toward paclitaxel sensitive and resistant cell lines. Yang et al. [129] reported doxorubicin-loaded AuNCgs for enhancing doxorubicin loading. The authors used a thermal effect on the release of the reported drug. As per the authors, in vitro studies showed the feasibility and advantage of the reported NPs for remote-controlled drug release systems. Sun et al. [130] developed doxorubicin-loaded AuNPs nano-conjugates for drug delivery. The drug release by NPs was better than free drug in MCF-ADR cells. As per the authors, well-designed drug delivery system and conventional chemotherapeutic agents may be promising for cancer stem cell therapy.

Li et al. [131] reported AuNPs PEGylated Polyamidoamine (PAMAM) dendrimers loaded with doxorubicin to improve therapeutic efficacy in in vitro and in vivo situations. The NPs showed uniform sizes and good colloidal stability. The drug release was pH controlled, and hence this may be used for controlled drug releases as per the requirement. The authors also demonstrated the combined photothermal-chemo treatment of cancer cells using PEG-DOX-PAMAM-AuNR for synergistic hyperthermia ablation. The chemotherapy was showed in both in vitro and in vivo conditions. Larson et al. [132] described AuNPs with aminohexylgeldanamycin (AHGDM), docetaxel and cisplatin drugs. The NPs were assessed in vitro for the ability to synergistically induce cytotoxicity in combination with moderate hyperthermia. The targeted docetaxel conjugates showed high potency against DU145 cells with an IC_50_ of 2.4 nM. Topete et al. [133] described the preparation of PLGA/DOXO-core Au-branched shell nanostructures (BGNSHs) functionalized with a human serum albumin/indocyanine green/folic acid complex (HSA-ICG-FA) to provide a multifunctional nanotheranostic platform. The prepared NPs were loaded with doxorubicin (topoisomerase II inhibitor and DNA intercalating agent). The reported formulation was tested by determining the biodistribution of the drug in a tumor bearing mice model. Hwu et al. [134] developed paclitaxel-PEG-SH coupled AuNPs for anticancer activities. Some other authors reported folic-acid-loaded PEGylated AuNPs for targeting purposes to cancerous cells [135,136,137]. The polymeric gold NPs used for drug delivery are given in Table 3.

### 4.5. Carbon Nanotubes (CNTs)

It is a well-known fact that the carbon nanotubes are the miracle identities in the world of nanoscience. The oxidation and grafting polymers on side walls of carbon nanotubes are useful to load the polymeric materials. In this series, polymeric materials have been conjugated with carbon nanotubes (CNTs). Feazell et al. [150] loaded DOX onto branched PL-PEG functionalized CNTs by π-π stacking to the sidewall of carbon nanotubes. In vivo studies in a mouse model of breast cancer indicated increased therapeutic efficacy and a marked reduction in toxicity as compared to free DOX. The same group [151] used paclitaxel to load on polymeric CNTs for testing cancer. Gao et al. [152] prepared nano/microbubbles of biodegradable block copolymers. These were found to be suitable for dramatical release of drugs. The authors reported suitable chemotherapy for MDA MB231 breast cancer. Chen et al. [153] prepared long polymer nanotubes with cross linked poly(glycidyl methacrylate) and a pendant poly(*N*-isopropyl acrylamide) for delivery of anti-cancer drugs. The authors used doxorubicin as a model drug for the study. The developed polymer nanotubes were found to have good biocompatibility in the test with KB-3-1 cancer cells. The toxicity assay confirmed IC_50_ value as 1.4 μmol L^−^^1^. It was observed that the described polymer nanotubes had a good potential for effective anti-cancer drug delivery.

Zhang et al. [154] developed multifunctional targeted poly(lactic-co-glycolic acid) (PLGA) nanobubbles (NBs) for use as targeted anticancer drug carriers and a synergistic agent for increasing therapeutic efficiency of High-intensity focused ultrasound (HIFU) ablation. The authors used methotrexate (MTX)-loaded NBs with active tumor-targeting monoclonal anti-HLA-G antibodies (mAbHLA-G) coated onto the surfaces of nanobubbles. These tubules were tested both in vitro and in vivo. The authors reported the augmented efficiency of HIFU ablation. It was observed that the reported tubules constituted an efficient and non-invasive HIFU synergistic treatment of cancer with the additional tasks of massacring remaining tumor cells and checking tumor reappearance/metastasis. Yang et al. [155] designed and developed triple stimuli-responsive, biodegradable nanocapsules having perfluorohexane and doxorubicin-loaded poly(methacrylic acid (PMAA) of 300 nm. It was observed that the drug loading efficiency was about 93.5%. It was observed that the reported nanocapsules may be biodegradable with a good therapeutic effect. Pistone et al. [156] reported two pH and temperature-precise drug delivery systems for cancer therapy by using hydrophilic polyethylene glycol (PEG) functionalized multi-walled carbon nanotubes. The NPs were loaded with doxorubicin and covered with the biocompatible polylactide and measured for drug release performance on three different human cancer cell lines. The authors evaluated and compared, stressing the opportunity to adjust the quantity of drug released by regulating the functionalization grade of the carbon nanotube material. The biological tests emphasized the high biocompatibility of polymeric carbon nanotubes to deliver DOX to cancer cells.

### 4.6. Polymeric Micelles

A micelle is an accumulation of surfactant molecules with both hydrophobic and hydrophilic parts, which undergo self-assembly in an aqueous media to form arrangements with a hydrophobic focus with a stable hydrophilic encapsulation. The beneficial features of polymeric micelles are their drug-carrying abilities, including solubilization of not-very-soluble drugs, and their abilities for tumor direction and precise drug discharge. The ease of micelle preparation by self-assembly of amphiphilic block copolymer moieties and drug encapsulation by physical entrapment rather than chemical conjugation are very pretty properties of polymeric micelles. These are collected by different means (outward from the core), including preparation of the monomer (G) in the main core [157] or convergent (inner core) methods [158]. Micelles are good identities in chemotherapy as colloidal transporters for hydrophobic drugs [97,159]. The small size of micelles is a good feature for penetrating cancer tissue for drug release. The micelles have been used to deliver low water solubility anticancer drugs to the target in animal models [160,161]. First of all, the polymeric micelles were introduced as drug delivery carriers in the early 1980s by Helmut Ringsdorf [162]. Besides, Genexol-PM formulation has got Food and Drugs Administration (FDA) consent for use in breast cancer treatment [95]. Some reviews have appeared on the use of micelles for drugs delivery [33,35].

Valenzuela-Oses et al. [163] developed a formulation of miltefosine-loaded polymeric micelles of the copolymer Pluronic-F127. The formulation was tested against HeLa carcinoma cells, which showed promising results. As per the thermal analyses, miltefosine was molecularly spread within PM. Pluronic-F127 polymeric micelles with miltefosine (80 μM) offered a noteworthy decrease of hemolytic effect (80%, *p* < 0.05) in contrast to free drugs. It was observed that miltefosine-loaded Pluronic-F127 polymeric micelles may be a hopeful nanocarrier in cancer therapy. Yang et al. [164] developed a polymeric on polyethylene glycol derivatized GA (PEG-Fmoc-GA) for DOX delivery. It was observed that this formulation had more effective synergistic action on cell proliferation inhibition and apoptosis. Besides, the formulation had long blood circulation time, drug concentration area under the curve and low distribution and clearance of DOX. As per biodistribution studies, the formulation was found to be accumulated at the tumor site. As per the authors, the so formed-polymeric prodrug micelles may be a promising dual-function co-delivery system to attain synergistic anti-cancer activity of DOX and GA.

Wang et al. [165] reported the improved blood retention and tumor accumulation of gambogic acid (GA) in vitro by encapsulating it in poly(ethylene glycol)-poly(ε-caprolactone)-poly(trimethylene carbonate) [MPEG-P(CL-ran-TMC)]. It was observed that the reported NPs indicated better dispersion in water, prolonged release behavior in vitro and enhanced tumor cellular uptake, showing superior antitumor efficacy and better apoptosis. Volsi et al. [166] reported the synthesis of polymeric micelles encapsulating both doxorubicin and gold core-shell quantum dots nanoparticles (Au-SiO_2_/QDs). The formulation was found to have the cytotoxicity of the breast cancer cells. The micellar NPs in clinical trials are shown in Table 4.

### 4.7. Dendrimers

The dendrimers are extremely branched synthetic polymers with symmetric centers and spherical 3-dimesional micro-molecules [176]. The dendrimers have five parts; i.e., a core, branches (called generations) emanating from the core, repeating units, at least one branch junction and numerous terminal functional groups. Generally, the center is made of diaminobutyl (DAB), ethylene diamine (EDA), polypropylimine (PPI) and polyamidoamine (PAMAM), with numerous exterior residues, such as carboxyl, amine and alcoholic functional groups, determining surface properties. Generally, a dendrimer is prepared with various materials depending on the properties required, such as dimension, shape, thermal stability, solubility, etc., using a convergent or divergent approach. These are changed as water-soluble my surface modifications. These modifications enhanced the capabilities of dendrimers in chemotherapy. These are classified by types of polymers (hyper branched and brush polymers) and their molecular weights (low and high molecular weight). Generally, dendrimer drug conjugations are made of covalent bonding between dendrimers and anticancer drugs. The melanin conjugated dendrimers were used to increase the solvability of anti-cancer drugs such as 6-mercaptopurine and methotrexate [177]. Highly branched dendrimer augmented aptamer probes may be simply reconstructed and have great appeal and specificity for a wide range of objectives. These are capable of reaching numerous targets with such great reliability, sensitivity and selectivity due to their good magnetic, optical, electric, biological and chemical properties [106]. The gold NP-based dendrimers have been used for the photothermal treatment of cancers [178,179]. Some reviews have been appeared on dendrimers drug delivery [36,38,180,181]. Malik et al. [182] reported PEGylated PAMAM dendrimer loaded cisplatin against B16F10 solid melanoma tumors. On the other hand, Kaminskas et al. [183] described PEGylated poly-L-lysine (PLL) dendrimers (G5, PEG1100) loaded with methotrexate as accumulated in HT1080 fibrosarcoma tumors in rats and mice.

Al-Jamal et al. [184] described doxorubicin-loaded cationic PLL dendrimer Gly-Lys63 (NH2)64 as anti-angiogenic active in tumor-bearing mice. Amreddy et al. [185] developed a folic acid (FA)-conjugated polyamidoamine dendrimer (Den)-based nanoparticle for co-delivery of siRNA against HuR mRNA (HuR siRNA) and cis-diamine platinum (CDDP) to folate receptor-α (FRA)-overexpressing H1299 lung cancer cells. The co-delivery of HuR siRNA and CDDP using the FRA-targeted NP had a meaningfully greater therapeutic effect than individual therapeutics. Besides, FRA-targeted NP showed improved cytotoxicity compared to non-targeted nanoparticles with lung cancer cells. The dendrimers and drugs interactions on cancer therapy are given in Table 5.

### 4.8. NPs Prepared by Electrospray

Boda et al. [192] reviewed the electrospraying as a versatile technology for manufacturing nanoparticles of various compositions, morphologies and sizes, which can be applied for imaging for diagnostics, drug delivery and sensing. Astolfi et al. [193] mentioned that cisplatin is a famous chemotherapeutic anticancer drug; however, the side effects, such as alopecia, nausea, nephrotoxicity and cardiotoxicity, limited the usage of this drug. Bai et al. [194] reported a simple method of preparing PEG-PLGA encapsulated cisplatin bioconjugated with CD44 antibody using electrospray. The anti-proliferative ability of the cis-encapsulated CD44-PEG-PLGA nanoparticles is 10%–14% greater against SKOV-03 and CP70 cells, which is useful in cancer therapy of targeting ovarian cancer cells in vivo or in vitro. Guo et al. [195] prepared core-shell polymer microparticles for theranostics by coaxial electrospray. In the microparticles, gold nanoparticles encapsulated folic acid conjugated chitosan as Au@CS-FA, and the shell was loaded with doxorubicin (DOX). The microparticles can generate strong surface-enhanced Raman scattering (SERS) signals for cancer detection due to highly branched gold nanoparticles inside. Therefore, as a laser-irradiated the microparticles, the locations of cancer cells could be imaged, and the DOX could be released due to the heat generated by the irradiating of Au@CS-FA particles simultaneously. Wu Y. [196] synthesized theranostic lipoplexes in one step by static micromixer-coaxial electrospray (MCE) technology with high reproducibility. QD605/Cy5-G3139-loaded lipoplexes were produced from the model imaging reagent quantum dots (QD605) and the anti-cancer drug Cy5-labelled antisense oligonucleotides (Cy5-G3139) by MCE. They were highly uniform with a polydispersity of 0.024 ± 0.006 and mean diameter by volume of 194 ± 15 nm. The lipoplex particles were efficiently delivered to A549 cells, a non-small cell lung cancer cell line, and the Bcl-2 gene expression could be down-regulated by 48% ± 6% simultaneously.

### 4.9. Nanoparticles Prepared by Supercritical Fluids

Taboada et al. [197] synthesized monodisperse iron oxide/microporous silica core/shell composite nanoparticles, core(g-Fe_2_O_3_)/shell(SiO_2_), in a one-step process with a high magnetization by combining sol-gel chemistry and a supercritical fluid method. These nanoparticles show superparamagnetic behavior at room temperature, are proven to be a suitable T2 MRI imaging agent and have the potential to be used for theranostic drug delivery. Chattopadhyay et al. [198] generated magnetically responsive poly(methyl methacrylate) using supercritical antisolvent (SAS) technology. These magnetite-encapsulated polymer nanoparticles can be used as contrast agents for magnetic resonance imaging scans for controlled-release drug formulations. The sizes of the nanoparticles are controllable via the SAS-EM technique developed at Auburn University, and the morphologies and drug-release kinetics of these nanoparticles were also studied. Chen et al. [199] prepared 5-fluorouracil-poly(L-lactide) (5-Fu-PLLA) microparticles by a solution-enhanced dispersion by supercritical CO^2^ (SEDS) process; the 5-fluorouracil is an anti-metabolite with activity against colon, rectal and prostate cancers. Therefore, the therapeutic efficacy of the 5-Fu drugs is increased by encapsulating the drugs in biodegradable polymers as sustained delivery systems. Chen et al. [200] synthesized indocyanine green (ICG)-encapsulated silk fibroin (SF) (ICG-SF) nanoparticles by supercritical fluid (SCF) technology. The encapsulated nanoparticles have excellent photothermal stability and can be observed with near-infrared (NIR) light at 808 nm. The ICG can be released from SF specifically due to this process being pH-responsive in the tumor acidic environment, and these nanoparticles can destruct cancer cells barely under light-induced hyperthermia in vitro and in vivo. These results showed that the biocompatible ICG-SF nanoparticles synthesized by SCF technology had high photothermal therapy efficiency, and showed the great potential of theranostic nanomedicine for cancer.

### 4.10. Miscellaneous Polymeric NPs

Besides the above-discussed polymeric NPs, some other biodegradable materials have been conjugated or prepared for loading and delivering drugs in cancer treatment. These include, chitosan, polystyrene, etc. Kim et al. [201] described chitosan-based nanoparticles (CNPs) and used them for control drug release. The authors described their unique features like deformability, stability and quick uptake by tumor cells. The loaded drug was paclitaxel and the reported NPs showed pointedly augmented tumor homing ability with low non-specific uptake by other cells tumor having mice. The authors reported that these NPs may be future anti-cancer agents. Park et al. [202] modified three glycol chitosan (GC—20 kDa, GC—100 kDa and GC—250 kDa) derivatives with cholanic acid to increase the in vivo tumor targeting features of polymeric nanoparticles. These amphiphilic glycol chitosan cholanic acid molecules conjugated self-assembled into glycol chitosan nanoparticles (GC—20 kDa NP, GC—100 kDa NP and GC—250 kDa NP). The distribution, tumor accumulation and time-dependent excretion of these NPs were determined in SCC7 tumor having mice using NIR fluorescence imaging systems. These NPs showed the most decreased time-dependent excretion and blood circulation time and raised tumor accumulation with growing polymer molecular weight. It was observed that high molecular weight glycol chitosan NPs remained for a longer time in the blood circulation, leading to an augmented gathering at the tumor site. The authors proposed that improved tumor targeting by high molecular weight NPS was related to better in vivo stability; centered on a pharmacokinetic enhancement in blood circulation period. Gao et al. [203] encapsulated doxorubicin in polymeric micelles. The materials used were pluronic P-105, PEG2000-diacylphospholipid and poly(ethylene glycol)-co-poly(beta-benzyl-L-aspartate) micelles as drug carriers. The authors compared drug bio-distribution with a molecularly dissolved one in ovarian carcinoma tumor model in mice. The authors reported good and controlled bio-distribution in the encapsulated formulation. Lammers et al. [204] used radionuclide (^131^I) with *N*-(2-hydroxypropyl)-methacrylamide (HPMA) polymer loaded with doxorubicin and gemicitabine for therapeutic efficiency. Dunning AT1 rat prostate carcinoma was utilized as a model for study. It was observed that the radioactive polymeric transferor indicated choosy gathering and lengthy movement period. The synergistic effect increased the therapeutic effects on cancer cells.

## 5. Polymeric NPs in Clinical Trials

During the last few years, some polymeric NPs have been tested in clinical trials while some others have been approved by FDA. The most important formulations are Genexol^®^-PM, NK105, NC-6004, NC-4016, NK012, NK911 and SP1049C. Doxil (pegylated liposomal doxorubicin) is the first nano-drug approved by FDA in 1995 to treat some cancers: AIDS-related Kaposi’s sarcoma, ovarian cancer, etc. The formulation contains doxorubicin (adriamycin) in about 80 nm size liposomes covered with PEG. This formulation allows the drug molecules to remain in the bloodstream for a long time for drug distribution to cancer cells to be prolonged [205,206]. For the approval of generic version of Doxil by FDA in 2003, Sun Pharma in India started its production at a large scale [207]. Another formulation is Lipodox made of doxorubicin and pegylated liposomes with a surface coating of PEG [208]. Some studies were carried out for clinical trial phases of DOX-loaded NPs [209].

**Figure 4:**
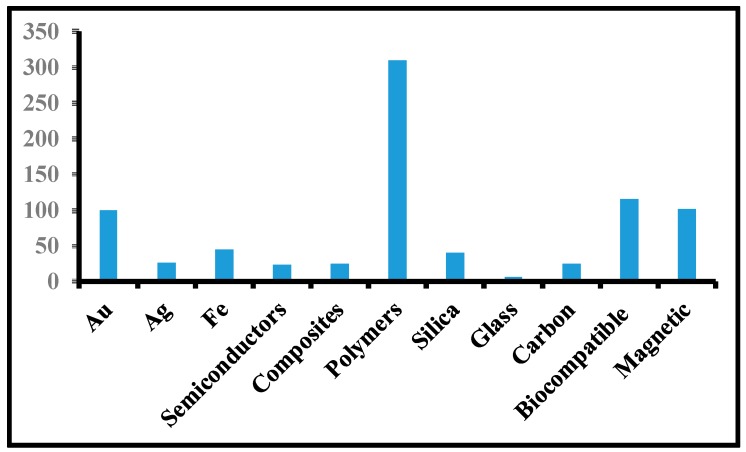
Numbers of the patents on the different types of NP-based drugs deliver systems.

Albumin nanoparticles (Abraxane^®^) have been approved by FDA for chemotherapy transportation for different types of cancer treatment [210,211]. Genexol-PM is approved by FDA for breast cancer treatment [98]. The polymeric micellar based drug (NK911) was tested under clinical trial in Japan in 2001. The formulation was having DOX encapsulated in PEO_5000_-b-P(Asp)_4000_-DOX micelles. After that, the formulation entered clinical trial phase II at National Cancer Center (NCC) Hospital in Japan. The proposed formulation was found to be effective in animal models but with some side effects [212]. Danson, Alakhov et al. [213] reported pluronic formulation with DOX (SP1049C) in an animal model, which was later entered in a clinical trial in Canada in 1999. In 2004. Kim et al. [214] reported PTX-loaded PEO2000-PDLLA1750 micelles (Genexol s-PM) in animal models. As per the authors, the maximum tolerated dose (MTD) of micellar PTX Genexols-PM was three times higher than of non-micellar formulation Taxols. PTX was loaded onto the polymeric micelles of PEG–P(Asp) modified with 4-phenyl-1-butanoate. This formulation was named NK105 [215], showing about 90 and 25 times higher plasma and tumor Area under the curve (AUCs), respectively, as compared to free PTX. This formulation NK105 is under phase I clinical trial at the National Cancer Center in Japan. Many patents have been filed by different workers globally. A comparison of patent filings is shown in Figure 4. It is clear from this figure that a maximum of 340 patents were filed on polymeric NPs, indicating the utility of polymer-based NPs. The liposomal NPs in clinical trials are given in Table 6.

Regarding the market status, about 30 nano-anticancer drugs are available in the market, with 17.5 billion USD made in 2011, 21.6 billion USD in 2012 and 53.7 billion USD in 2017; the figure is expected to reach 136 billion USD in 2021. This increase is due to the development of diagnostic instruments, detail patient information and the approval of several drugs by the FDA and other drugs controlling agencies globally. Some famous companies working on the production of nano drugs are Access Pharmaceuticals, ProLindac, Leonardo Bio Systems, Abraxis Biosciences, Immunolight LLC, Abbot and Sun Pharma. The approved polymeric nanomedicines for cancer therapy are given in Table 7.

## 6. Merits and Demerits of Polymeric NPs

The most important advantages of polymeric NPs are their small sizes, biocompatibility, biodegradability, high drug payload, low or no toxicity to normal (healthy) cells, high stability in physiological conditions, capacity to carry the imaging agents, controlled release of drugs, NPs-drug complex stability at biological buffered pH, NPs-drug complex receptor facilitated interfaces, etc. Besides them, other advantages include ease of their preparation and good control over size and distribution. The polymeric NPs have good protection capabilities for the encapsulated drugs regarding environmental factors. The good drug retention and long blood circulation times are other assets of polymeric NPs. There are no serious demerits of polymeric NPs. However, the limited targeting abilities and the problem of discontinuation of the therapy may be considered the major drawbacks. Sometimes, the autonomic imbalance disturbance may occur via polymeric NPs. Occasionally, polymeric NPs show effects on vascular and heart functions.

## 7. Future Perspectives

Of course, polymeric NPs are successful candidates for cancer detection, treatment, and management. Not to limit them to cancer chemotherapy, these NPs have been also exploited for AIDS, radiation, and gene therapies. The future is quite bright for these NPs in delivering antibiotics, proteins, vaccines, and virostatics to various cells. Despite good progress in cancer diagnosis, still, the techniques are not advanced enough to detect cancer at early stages. Moreover, the sensitivity and selectivity of conventional diagnostic techniques are not quite enough. Additionally, some toxicity has been reported due to the use of polymeric NPs. However, these NPs are the hope for a wide range of platforms for a varied array of biomedical applications in the future.

It is important to mention here that polymeric NPs are not fully developed and need more advances. The complex structures of the polymeric NPs used are difficult to handle during preparation. The imaging strategies need to be improved to avoid toxicity and advancement in anatomical to molecular imaging. Besides, it is essential to modify the physical, chemical, and biological properties to increase biocompatibility, biodegradability, and drug loadability; reduce toxicity to low levels or none for normal cells; and extend circulation time. The identification of the requirements of the specific combinations of NPs carriers and target molecules is very important. Certainly, efficiency enhancements will lead to improved therapeutic results and low toxicity. Also, there is a need to find out the pharmacotherapy with special emphasis on absorption, distribution, degradation, and excretion mechanisms in blood. There are certain issues to be tackled by PNPs, such as low drug solubility, the differences between loading and tracking of the drugs; non-selective tissue penetration, and the drug binding interferences. Finally, the pre-clinical trials, clinical trials, and the approval time period should be curtailed to provide cancer medication to the patients.

## 8. Conclusions

Nano-theranostics is a hopeful approach to cancer diagnosis and treatment under one umbrella. The polymeric nanoparticles have been synthesized and used for diagnosis and drugs delivery. It is only possible to provide all the three aspects of cancer cure (diagnosis, treatment, and management) by polymeric NPs. Theranostics also provide a holistic transition from trial and error medication to personalized medication. The polymeric NPs are the only safe chemotherapeutic anticancer drugs carriers. Some polymeric nanoparticle-based nanodrugs are approved by the sanction agencies while some others are in clinical trials. It is expected that the polymeric nano drugs are future medications to eradicate cancer from its root. Certainly, this area of research promises to augment the excellence of clinical care and treatments. Besides, the use of polymeric nanodrugs will ultimately save the lives of millions and billions of USD in the future. But still, there is a great need to develop these drugs collectively at the global level.

## Figures and Tables

**Table 1:** The biocompatible polymer-based cancer theranostics.

Imaging Modality	Polymers	Imaging Agents	Encapsulated Moiety	Targeting Moiety	Refs
MRI	Poly(trimethylene carbonate)–poly(L-glutamic acid) (PTMC-b-PGA copolymer)	Ultrasmall superparamagnetic iron oxides (USPIOs)	Doxorubicin		[51]
MRI	PTMC-b-PGA copolymer	USPIOs	Doxorubicin	HER-2	[52]
MRI	Poly(acrylic acid-co distearin acrylate)	Superparamagnetic iron oxide nanoparticles (SPIONs)	Doxorubicin	Folic acid	[53]
MRI	PluronicR L-121 (EO5-PO68-EO5)	SPIONs	Camptothecin	Bombesin	[54]
MRI	PTMC b-PGA copolymer	USPIO’s	Doxorubicin		[55]
MRI	(FA or methoxy)-PEG114–P (Glu-Hyd-DOX)-PEG–acrylate46 SPIO	Superparamagnetic iron oxides (SPIO)	Doxorubicin	Folic acid	[56,57]
MRI	PEO–PLGA–PEO (polyethylene oxide–poly D,L lactide-co-glycolide–polyethylene oxide)	Iron oxide (Fe_3_O_4_)	Doxorubicin		[58]
MRI	methoxy or folate (FA)-PEG 114–PLA-PEG-acrylate 46 SPIO NPs	SPIO NPs	Doxorubicin	Folic acid	[56,57]
Ultrasound	PLGA (poly(D,L-lactide-coglycolide))	Fe_3_O_4_	H2O2		[59]
Ultrasound Optical imaging	Poly(ethylene glycol)-bpoly(L-aspartic acid) (PEG-PAsp)	-	Doxorubicin Calcium carbonate	Rabies virus glycoprotein (RVG) peptide	[60]
Optical imaging	PLGA	Mercaptosuccinic acid( MSA)-coated CdTe QDs	Doxorubicin		[61,62]
Optical imaging	poly(2-hydroxyethyl-cooctadecyl aspartamide) (PHEA-C18)	Magnetic resonance tomography (MIR) fluorescent probe, FPR-675	Folic acid		[63]
Optical imaging	poly(ethylene glycol)-copoly(trimethylene carbonate-cocaprolactone)	Multiporphyrin near-infrared (NIR) fluorophores			[64]
Optical imaging	PPF–PLGA–PEG polymer	Rhodamine B	Folic acid		[65]
Optical imaging	PPF–PLGA–PEG copolymer	-	Doxorubicin		[66]
Optical imaging	Polytyrosine	-	Doxorubicin		[67]
Optical imaging	Polyethylenimine	Hematoporphyrin	-		[68]

**Table 2:** The polymer NP-based drugs in clinical trials.

Products	Drugs	Platform (NPs)	Status	Applications	Refs
Abraxane	Paclitaxel	Albumin	Approved	Lung, breast and pancreatic cancers	[69]
ABI-009	Rapamycin	Albumin	Phase I/II	Solid tumors	[70]
ABI-008	Docetaxel	Albumin	Phase I/II	Prostrate & breast cancers	[71]
BA-003	Doxorubicin	Polymeric	Phase III	Hepatocellular cancer	[72]
BIND-014	Docetaxel	PEG-PLGA polymeric	Phase I/II	Lung cancer	[73]
CALAA-01	siRNA targeting	Cyclodextrin	Phase I	Solid tumors	[74]
DHAD-PBCA-NPs	Mitoxantrone	Polymeric	Phase II	Hepatocellular cancer	[75]
Docetaxel-PNP	Docetaxel	Polymeric	Phase I	Solid tumors	[76]
Nanotax	Paclitaxel	Polymeric	Phase I	Neoplasms	[77]
ProLindac	DACHPt	HPMA-polymeric	Phase II/III	Ovarian cancer	[78]

**Table 3:** The polymeric gold NPs as carriers in drug delivery.

Au NP Modifications	Drugs Loaded	Functionalization Modes	Cell Lines and Models	Refs
Aminated dextran	Doxorubicin	Covalent linking	HeLa cells	[138]
Folate altered and PEG-SH	Docetaxel	Physical sorption	LnCaP cells	[139]
Folate adapted PEG	Doxorubicin	Physical sorption	KB cells	[140]
Folic acid & PEG-SH	Cisplatin	PEG-SH linking	OVCAR-5, OV-167, HUVEC, OSE	[141]
Folate & polyacrylamide	Methotrexate	Covalent linking	KB cells	[142]
PEG-folate	Curcumin	Hyaluronic acid (HA)-curcumin linking	HeLa cells, C6 glioma cells and caco 2 cells	[143]
PEG-SH	Oxaliplatin	PEG-SH linking	A549, HCT116, HCT15, HT29, RKO	[144]
PEG-SH	Tamoxifen	PEG-SH linking	MDA-MB-231, MCF-7, HSC-3	[145]
PEG bis amine & mercaptopropionic acid	Doxorubicin	DOX-SH via disulfide linking	MDR cells	[146]
PEG	Paclitaxel	Paclitaxel-PEG-SH linking	HepG2 cells	[128]
PEG-SH, cyclodextrin as drug pocket, anti-EGFR	B-Lapachon	Physical sorption	OV-167, OVCAR-5, HUVEC, OSE MCF-7	[137,147]
PEG-SH and methyl thioglycolate	Doxorubicin	PEG-SH linking	4T1 cells	[148]
TNF & PEG–SH	Paclitaxel	Paclitaxel-SH linking	MC-38, C57/BL6 mice implanted with B16-F10 melanoma cells	[149]

**Table 4:** The micellar NPs based drugs in clinical trials.

Products	Drugs	Platform	Status	Applications	Refs
Genexol-PM	Paclitaxel	mPEG-PLA polymeric micelle	Approved	Breast cancer	[167]
Lipotecan	Camptothecin analog	Polymeric micelle	Phase I/II	Renal & liver cancers	[168]
NC-6004	Cisplatin	PEG-PGA polymeric micelle	Phase II/III	Genitourinary & gastrointestinal cancers	[169]
NK012	SN-38	PEG-PGA polymeric micelle	Phase II	Lung, colorectal & ovarian cancers	[78]
NK105	Paclitaxel	PEG-PAA polymeric micelle	Phase II/III	Gastric & breast cancers	[170]
NC-6300	Epirubicin	PEG-*b*-PAH polymeric micelle	Phase I	Solid tumors	[171]
NC-4016	Oxaliplatin	Polymeric micelle	Phase I	Solid tumors	[172]
Paclical	Paclitaxel	Polymeric micelle	Phase III	Ovarian cancer	[173]
NK911	Doxorubicin	PEG-PAA polymeric micelle	Phase I	Solid tumors	[174]
SP1049C	Doxorubicin	Pluronic L61 and F 127 polymeric micelle	Phase II/III	Lung cancer	[175]

**Table 5:** The dendrimers and drugs interactions in cancer therapy.

Dendrimers Used	Drugs Used	Drug-Dendrimer Interactions	Functions	Refs
Carboxylated PAMAM Dendrimer G3.5	Cisplatin	Covalent Conjugation	Good loading efficiency & reduced cytotoxicity	[186]
Cyclic arginine-glycineaspartic acid (RGD) peptide-conjugated generation 5 (G5) poly(amidoamine) dendrimers	Doxorubicin	Encapsulation	Drugs released in a sustained way	[187]
56 kDa PEGylated polylysine dendrimer	Doxorubicin	Covalent Conjugation	Inhalable drug delivery systems	[188]
Omega-3 fatty acid [docosahexanoic acid (DHA)]-poly(amido)amine (PAMAM) dendrimer	Paclitaxel	Covalent Conjugation	Good anticancer activity	[189]
6 kDa and 20 kDa Polyethylene glycol dendrimer	Paclitaxel	Covalent Conjugation	Long drug residential time & sustained-release with reduced toxicity	[190]
Hialuronic acid-amine terminated fourth generation poly(amidoamine) (PAMAM) dendrimer	3,4-Difluorobenzylidene Curcumin	Encapsulation	Targeted specificity and good cellular uptake	[191]

**Table 6:** The liposomal NPs based drugs in clinical trials.

Products	Drugs	Status	Applications	Refs
Aroplatin	Cisplatin	Phase II	Colorectal cancer	[216]
Atragen	Tretinoin	Phase II	Leukemia	[217]
Atu027	PKN3 siRNA	Phase II	Solid tumors	[218]
CPX-351	Cytarabine & daunorubicin	Phase III	Myeloid leukemia	[219]
Anti-EGFR immuno-Doxorubicinliposomes	Doxorubicin	Phase I	Solid tumors	[220]
Doxil	Doxorubicin	Approved	Ovarian and breast cancers & Kaposi sarcoma	[221,222]
EndoTAG-1	Paclitaxel	Phase II	Pancreatic & breast cancers	[223]
INX-0125 V	Vinorelbine	Phase I	Solid tumors	[224]
INX-0076	Topotecan	Phase I	Solid tumors	[225]
LipoDox	Doxorubicin	Approved	Ovarian and breast cancers	[226]
LEP-ETU	Paclitaxel	Phase II	Breast, ovarian & lung cancers	[227]
LE-SN38	SN38	Phase II	Colorectal cancer	[228]
Lipoplatin	Cisplatin	Phase III	Breast, pancreatic, neck & head, gastric, and non-squamous non-small cell lung cancers	[229]
Liposomal annamycin	Annamycin	Phase I/II	Lymphocytic leukemia	[230]
Liposomal Grb-2	Grb2-antisenseoligodeoxynucleotide	Phase I	Myeloid leukemia, chronic myelogenous leukemia, & acute lymphoblastic leukemia	[231]
LEM-ETU	Mitoxantrone	Phase I	Breast, stomach, leukemia, breast, stomach, liver, and ovarian cancers	[232]
LiPlaCis	Cisplatin	Phase I	Refractory tumors	[233]
Lipoxal	Oxaliplatin	Phase I	Gastrointestinal cancer	[234]
Myocet	Doxorubicin	Approved	Breast cancer	[235]
Marqibo	Vincristine	Approved	Uveal cancer	[236]
MBP-426	Oxaliplatin	Phase II	Gastroesophageal, gastric & esophageal adeno-carcinomas	[237]
Onivyde	Irinotecan	Approved	Pancreatic cancer	[238]
OSI-211	Lurtotecan	Phase II	Head, ovarian & head and neck cancers	[239]
OSI-7904L	Thymidylate synthaseinhibitor	Phase I/II	Colorectal, head & neck, gastric, and gastroesophageal cancers	[240]
Stimuvax	BLP25 Tecemotide	Phase III	Multiple myeloma developed encephalitis	[241]
SPI-077	Cisplatin	Phase II	Ovarian and head and neck cancers	[242]
ThermoDox	Doxorubicin	Phase III	Non-resectable hepatocellular cancer	[243]

**Table 7:** The approved polymeric nanomedicines for the cancer therapy.

Drugs Used	Polymers	Manufacturer	Cancers	Approval Status
Abraxane	Paclitaxel in combination with gemcitabine & albumin-bound Paclitaxel nanospheres	Astrazeneca Celgene & Abraxis Bioscience	Metastatic pancreatic cancer	Approved in September 2013
Aroplatin	Liposomes	Antigenics, Inc.	Colorectal cancer	In clinical phase I/II
Atragen	Liposomes	AronexPharmaceuticals	Promyelocyticleukemia	In clinical phase II
Aurimmune (CYT-6091)	TNF-α	Cytimmune Sciences	Neck & head cancers	In clinical phase II
Cisplatin	PEG-b-poly(L-glutamic acid)	Nanocarrier, Co., Japan	Pancreatic cancer	In clinical trial phase II
Doxil (Caelyx)	Pegylated	Orthobiotech, Schering-Plough	Breast & ovarian cancers	Approved in 1995
Doxorubicin	Liposomes	-	Ovary cancer	In clinical trial, Jan., 2008, Russia
Doxorubicin	PEG-b-poly(α,β-aspartic acid)	Nippon Kayaku, Co., Japan	Solid tumors	In clinical trial phase II
DepoCyt	Liposomes	Skye Pharma, Enzon	Lymphomatous meningitis	Approved in 1999
DaunoXome	Liposomes	Gilead Science	HIV-related Kaposi sarcoma	Approved in 1996
Epirubicin	PEG-b-poly(aspartate-hydrazone)	Nippon Kayaku, Co., Japan	Solid tumors	In clinical trial phase I
Genexol-PM	Polymeric micelle	Samyang	Lung & breast cancers	Marketed in Europe, Korea
LEP-ETU	Liposomes	Neopharma	Breast, lung & ovarian cancers	In clinical phase I/II
Lipoplatin	Liposomes	Regulon	Head, neck & pancreatic cancers	In clinical phase III
Myocet	Liposomes	Elan Pharmaceuticals/Sopherion Therapeutics	Breast cancer	Approved in 2000, Europe and Canada
Narekt-102	PEGylated liposome	Nektar Therapeutics	Colorectal & breast cancers	In clinical Phase 3
Onco-TCS	Liposomes	Inex	Non-Hodgkin Lymphoma	In clinical phase I/II
OSI-211	Liposomes	OSI	Ovarian & lung cancers	In clinical phase II
Oncaspar	PEGasparaginase	Enzon	Lymphocytic Leukemia	Approved in 1994
Ontak	Diphtheria toxin and	Seragen, Inc.	Cutaneous T-cell	Approved 1999
Oxoplatin	PEG-b-poly(L-glutamic acid)	Nippon Kayaku, Co., Japan	Solid tumors	In clinical trial phase I
Paclical	Paclitaxel micelles	Oasmia Pharmaceutical AB	Ovarian cancer	Phase III
Paclitaxel	Micelles	-	Breast cancer	In clinical trial, 2009, South Korea
Paclitaxel	PEG-b-poly(α,β-aspartic acid) PEG–poly(D,L-lactide)	Nippon Kayaku, Co., Japan	Bresat & gastric cancers	In clinical trial phase III
SPI-77	Stealth Liposomal Cisplatin	Alza	Neck, lung & head cancers	In clinical Phase III
ThermoDox	Doxorubicin	Celsion Corporation	Hepatocellular carcinoma	In clinical phase III
